# Predictability of Orthodontic Space Closure Using Invisalign Clear Aligners: A Retrospective Study

**DOI:** 10.7759/cureus.56706

**Published:** 2024-03-22

**Authors:** Mohammed A Barashi, Raneem M Habis, Hesham A Alhazmi

**Affiliations:** 1 Preventive Dentistry, Umm Al-Qura University, Mecca, SAU; 2 Dentistry, Umm Al-Qura University, Mecca, SAU

**Keywords:** accuracy, spacing, refinement, clincheck, invisalign

## Abstract

Introduction

Clear aligners have demonstrated success in achieving complex tooth movements. However, orthodontists have faced challenges related to the predictability of clear aligners. This retrospective study aimed to assess the predictability of ClinCheck® accuracy in space closure before and after Invisalign® treatment and to identify factors associated with the need for refinement.

Methods

Patient records from one private clinic in Makkah, Saudi Arabia, were analyzed, and a sample of 55 adult patients who had spacing and underwent Invisalign treatment were included. Data on demographic and orthodontic variables were collected, and a chi-square test was conducted to examine the association between the requirement for refinement and demographic as well as clinical/orthodontic factors. Furthermore, the initial and final space measurements were compared using paired t-tests across various demographic and clinical/orthodontic variables.

Results

After completing the treatment, 70.9% (N=39) of the cases did not require any orthodontic refinement. The mean final space measurement was higher for males compared to females (0.7 mm and 0.4 mm, respectively), individuals who received treatment in the upper compared to lower arch (0.5 and 0.4 mm, respectively), those with moderate compared to mild spacing (0.5 and 0.1 mm, respectively), and those with class III compared to class I Angle classification (0.9 and 0.3 mm, respectively). Additionally, patients with severe spacing had a significantly higher probability of requiring refinement compared to patients with mild spacing (adjusted odds ratio = 20.9; p < 0.05).

Conclusion

The study emphasizes the significance of careful patient selection and treatment planning, suggesting that orthodontists should consider overcorrecting in space closure when using clear aligners, especially in cases with more significant spacing.

## Introduction

Clear aligners produced by computer-aided design/computer-aided manufacturing (CAD-CAM) systems are thermoplastic removable orthodontic polymeric appliances used to achieve orthodontic movements [1]. The initial Invisalign® system (Align Technology, Inc., Tempe, AZ) introduced a sequence of transparent aligners that were individually designed to fit over the teeth, constructed from a thermoplastic material, and intended to gradually reposition the teeth throughout the treatment period. These aligners have gained popularity among patients due to their cosmetic appeal and clinical efficacy [2]. In comparison to conventional orthodontic appliances, clear aligners have been associated with improved periodontal health, better oral hygiene, and reduced oral bacterial levels [3-4]. Additionally, clear aligners have shown potential in minimizing the risk of root resorption and reducing the initial discomfort associated with orthodontic treatment [5-6]. However, there was no difference between conventional orthodontic appliances and clear aligners regarding gingival health when followed up by a dental hygienist [7].

Initially, clear aligners were primarily recommended for mild malocclusions, such as cases of mild crowding or space closure [8]. However, advancements in biomechanics have allowed for the treatment of more complex malocclusions, including class II and class III cases [9-10]. Clear aligners have also shown promising results in achieving complex tooth movements, such as rotation or torque [11]. Despite these advancements, orthodontists have encountered challenges regarding the predictability of clear aligners in certain types of tooth movements, often requiring multiple adjustments during treatment [12].

One of the most important functions of aligners is space closure, particularly with the use of the ClinCheck® tool from Align Technology Inc., Santa Clara, CA, which allows orthodontists to plan incisor torque control and molar anchoring to close extraction spaces, reduce crowding, and retract anterior teeth [13]. However, several studies have reported discrepancies between the predicted tooth crown movement in ClinCheck and the actual achieved tooth movement in both extraction and non-extraction treatments [14-16]. Loss of incisor torque has been observed in nearly 50% of patients, and variations between predicted and achieved tooth movement have been reported across different types of teeth [15,17]. Another study conducted by Kravitz et al. observed an accuracy rate of approximately 41% when comparing predicted and achieved tooth movement in 37 patients and 401 teeth treated with clear aligners [16]. On the other hand, Align Technology's internal research suggests that approximately 80% of the predicted tooth movement should be visible in ClinCheck [18]. Another study conducted in Saudi Arabia by Alswajy et al. evaluated the predictability of tooth movement using Invisalign in 206 patients from three private clinics, reporting a mean predicted accuracy of 76.9% [19].

Despite the widespread use of Invisalign and ClinCheck, limited data exist regarding the predictability of ClinCheck accuracy, particularly as newer generations of Invisalign have introduced advancements in space closure techniques [20]. Therefore, this study aims to assess the predictability of ClinCheck accuracy in space closure before and after Invisalign treatment.

## Materials and methods

Ethical consideration

Ethical approval was obtained from the College of Dentistry at Umm Al-Qura University Institutional Review Board (Ethics Approval No: HAPO-02-K-012-2023-11-1879).

Study design and study setting

This retrospective study used available patient records from a private clinic in Makkah, Saudi Arabia, where orthodontic treatment was performed using Invisalign clear aligners.

Inclusion and exclusion criteria

Healthy adult patients aged 18 to 60 years were recruited for the study. The candidates selected for the study had to meet the following criteria: (a) they had undergone orthodontic treatment with Invisalign clear aligners and (b) they were treated with either non-extraction with spaces or premolar extraction. Conversely, cases treated with conventional orthodontic appliances and cases without spaces were excluded.

Variables

The study had two primary outcomes. The first outcome was to determine whether the cases required orthodontic refinement by adding more aligners to achieve the original treatment goals after completing the treatment. The second outcome was to assess the amount of space remaining after orthodontic treatment, measured in millimeters (mm), and classified as mild (1-3 mm), moderate (3-5 mm), or severe (≥6 mm). The study included several demographic variables (age and gender) and clinical/orthodontic variables (length of treatment, arch type, and Angle classification) for analysis.

Sampling technique and sample size calculation

The sampling technique used in this study was convenience sampling, and the sample size consisted of 55 patients. The determination of this sample size was based on several assumptions. These assumptions included an average lack of correction of 50%, a difference of 0.5 as reported in a previous study, a test power of 90%, an α-level of 0.05, and an assumed standard deviation of 1 [21].

Data collection

Data collection was conducted at private practices where a single orthodontist, who was also an Invisalign provider, conducted the treatment plans. Patients’ data that met the inclusion criteria were obtained using the iTero intraoral scanner (Align Technology Inc., San Jose, CA), and the dental models were uploaded to the ClinCheck Pro software (Align Technology Inc.). Pretreatment and predicted posttreatment dental models were exported from ClinCheck. The pre-treatment space, predicted space, space closure, and final achieved space closure were measured separately for each arch.

Statistical analysis

Descriptive statistics were conducted for the demographic and clinical/orthodontic characteristics of both the entire patient sample and those who required refinement. The chi-square test was performed to assess the association between the need for refinement and the demographic and clinical/orthodontic findings. The mean initial space and final space measurements were compared for each category across different demographic and clinical/orthodontic variables using paired t-test. Furthermore, logistic regression analysis was performed to assess the association between the need for refinement and spacing status of the teeth. The model was controlled for age and gender, and adjusted odds ratios (AORs) were reported. All statistical analyses were conducted at a significance level of 5% (α = 0.05). The statistical analyses were performed using Stata Version 18 (StataCorp, College Station, TX, USA).

## Results

The majority of the patients were female (83.6%; N=46), below the age of 40 years (89%; N=49), underwent a treatment duration between six and less than nine months (52.7%; N=29), received treatment in the upper arch (58.2%; N=32), and had mild spacing (56.4%; N=31). Around 43% (N=24) of the patients had a class I Angle classification, while 36.4% (N=20) had a class II Angle classification. Overall, 29.1% (N=16) of the patients required refinement after completing the orthodontic treatment. Among those who needed refinement, a higher proportion was observed among patients aged 30-39 years compared to <30 years (45.8%; N=11 and 20%; N=5, respectively), those with a treatment duration of nine months or more compared to less than six months (62.5%; N=5 and 22.2%; N=4, respectively), those who received treatment in the upper compared to lower arch (31.3%; N=10 and 26.1%; N=6, respectively), those with severe compared to mild crowding (71.4%; N=5 and 12.9%; N=4, respectively), and those with a class II compared to class III Angle classification (40.0%; N=8 and 25.5%; N=6, respectively). A statistically significant association was found only between age, spacing (mild, moderate, or severe), and the likelihood of requiring refinement after completing orthodontic treatment (p < 0.05) (Table 1).

**Table 1:** Demographic and clinical/orthodontic findings among total patients and those requiring refinement ^a^Chi-square test. *Statistically significant (p < 0.05) N, number of participants

Demographic/orthodontic variables	Total	Need refinement		P-value^a^
	N=55	Yes, N=16 (29.1%)	No, N=39 (70.9%)	
Gender	N (%)	N (%)	N (%)	
Male	9 (16.4%)	4 (44.4%)	5 (55.6%)	0.27
Female	46 (83.6)	12 (26.1%)	34 (73.9%)	
Age				
<30 years	25 (45.5%)	5 (20%)	20 (80%)	0.03^*^
30-39 years	24 (43.5%)	11 (45.8%)	13 (54.2%)	
≥40 years	6 (11.0%)	-	6 (100%)	
Length of treatment				
<6 months	18 (32.7%)	4 (22.2%)	14 (77.8%)	0.11
6 months to <9 months	29 (52.7%)	7 (24.1%)	(75.9%)	
≥9 months	8 (14.6%)	5 (62.5%)	3 (37.5%)	
Arch				
Upper	32 (58.2%)	10 (31.3%)	22 (68.8%)	0.68
Lower	23 (41.8%)	6 (26.1%)	17 (73.9%)	
Spacing				
Mild	31 (56.4%)	4 (12.9%)	27 (87.1%)	<0.01^*^
Moderate	17 (30.9%)	7 (41.2%)	10 (58.8%)	
Severe	7 (12.7%)	5 (71.4%)	2 (28.6%)	
Angle classification				
Class I	24 (43.6%)	6 (25.0%)	18 (75.0%)	0.37
Class II	20 (36.4%)	8 (40.0%)	12 (60.0%)	
Class III	11 (20.0%)	2 (18.8%)	9 (81.8%)	

Table 2 presents the differences in initial space and final space across different demographic and orthodontic variables. Among all patients, the mean initial space was 3.3 mm, while the mean final space was 0.4 mm, with a statistically significant difference (p < 0.05). Males showed a higher mean initial space compared to females (5.4 mm and 2.9 mm, respectively), as did individuals aged 30-39 years compared to ≥40 years (4.1 mm and 2.2 mm, respectively), those who underwent treatment for nine months or longer compared to less than six months (7 mm and 2.0 mm, respectively), received treatment in the lower arch compared to upper arch (3.7 mm and 3.0 mm, respectively), had severe spacing compared to mild spacing (10.3 mm and 1.0 mm, respectively), and had a class III compared to class I Angle classification (4.4 mm and 2.8 mm, respectively). Conversely, males had a higher mean final space compared to females (0.7 mm and 0.4 mm, respectively), as did individuals aged 30-39 years compared to those aged 40 years or older (0.7 mm and 0 mm, respectively), those who underwent treatment for nine months or more compared to those who had treatment for less than six months (1.3 mm and 0.2 mm, respectively), received treatment in the upper arch compared to the lower arch (0.5 mm and 0.4 mm, respectively), had severe compared to mild spacing (2.0 mm and 0.1 mm, respectively), and had a class III compared to class I Angle classification (0.9 mm and 0.3 mm, respectively). The observed differences in initial and final space were statistically significant for all demographic and orthodontic variables (p < 0.05).

**Table 2:** Comparison of initial and final space measurements by demographic and orthodontic variables ^a^Paired t-test. *Statistically significant (p < 0.05) Mean initial and final spaces are given in millimeters SD, standard deviation

Demographic/orthodontic variables	Initial space, mean (SD)	Final space, mean (SD)	P-value^a^
Total	3.3 (3.1)	0.4 (0.9)	<0.01^*^
Gender			
Male	5.4 (2.9)	0.7 (0.9)	<0.01^*^
Female	2.9 (3.4)	0.4 (0.9)	<0.01^*^
Age			
<30 years	2.8 (2.7)	0.3 (0.8)	<0.01^*^
30-39 years	4.1 (4.1)	0.7 (1.1)	<0.01^*^
≥40 years	2.2 (2.6)	0	0.031^*^
Length of treatment			
<6 months	2.0 (1.9)	0.2 (0.3)	<0.01^*^
6months to <9 months	3.1 (2.7)	0.4 (0.8)	<0.01^*^
≥9 months	7 (5.6)	1.3 (1.5)	<0.01^*^
Arch			
Upper	3.0 (3.4)	0.5 (0.8)	<0.01^*^
Lower	3.7 (3.5)	0.4 (1.0)	<0.01^*^
Spacing			
Mild	1.0 (0.9)	0.1 (0.2)	<0.01^*^
Moderate	4.6 (1.4)	0.5 (0.7)	<0.01^*^
Severe	10.3 (3.0)	2.0 (1.5)	0.02^*^
Angle classification			
Class I	2.8 (2.6)	0.3 (0.7)	<0.01^*^
Class II	3.3 (2.8)	0.6 (0.5)	<0.01^*^
Class III	4.4 (5.5)	0.9 (1.6)	<0.01^*^

After controlling for gender and age, patients who had moderate spacing had higher probabilities of needing refinement compared to patients who had mild spacing (AOR= 4.9; p < 0.05). Additionally, patients who had severe spacing had higher probabilities of needing refinement compared to patients who had mild spacing (AOR= 20.9; p < 0.05) (Table 3). 

**Table 3:** Logistic regression analysis for patients who needed refinement by the spacing status of the teeth ^a^Adjusted for age and gender. *Statistically significant (p < 0.05).

Variables	Reference group	Adjusted odds ratio^a^	P-value
Moderate spacing	Mild spacing	4.9	0.038^*^
Severe spacing	Mild spacing	20.9	0.005^*^

## Discussion

The objective of this retrospective study was to assess the predictability of ClinCheck accuracy in achieving space closure before and after Invisalign treatment in a private clinic in Saudi Arabia. The study focused on patients aged between 18 and 60 years. The results showed that clear aligners demonstrated an accuracy of 70.9% (N=39) in achieving space closure. Previous studies have also examined the accuracy of clear aligners in tooth movement. Kravitz et al. evaluated in a prospective clinical study the efficacy of tooth movement with Invisalign for 37 patients. The authors measured the tooth movement by superimposing the virtual model of the predicted tooth position over the virtual model of the achieved tooth position. They reported a mean accuracy of 41% in anterior tooth movement [16]. Haouili et al. assessed the predictability of arch expansion using Invisalign. The authors included 64 patients and measured the accuracy of the treatment by creating pre- and posttreatment digital models. They found an overall mean accuracy of 50% for Invisalign in various tooth movements [22]. There are several factors that may have contributed to the higher accuracy percentage of space closure observed in our study. Firstly, all treatments were provided by a single American Board-certified orthodontist. Secondly, the inclusion criteria for case selection in this study may have contributed to better results compared to previous studies. However, it is important to note that longer treatment times in our study were associated with an increased likelihood of deviations from the predicted tooth position. These findings suggest that careful monitoring and adjustment of treatment plans may be necessary to ensure optimal outcomes when using clear aligners.

In our study, we observed several factors that were associated with higher mean final space measurements. These factors included being male compared to female, being in the age group of 30-39 years compared to those aged 40 years or older, having a treatment duration of nine months or more compared to those with less than six months of treatment, receiving treatment in the upper arch compared to receiving treatment in the lower arch, having severe spacing compared to in mild spacing, and having a class III Angle classification compared to Class I classification. Our results align with a prior systematic review that evaluated the clinical efficacy of clear aligner treatment in comparison to fixed appliance treatment. The review indicated that clear aligners demonstrated greater effectiveness in addressing mild-to-moderate malocclusion cases as opposed to severe cases. Additionally, the review showed that fixed appliance treatment exhibited shorter duration times for severe cases requiring extractions, in contrast to clear aligner treatment. In conclusion, the review suggested that fixed appliance treatment outperforms clear aligners in cases necessitating severe rotation, extrusion, and bodily tooth movement [5]. However, due to the relatively small sample size of males in our study, the difference between males and females should be taken with caution.

Our results showed that patients with severe spacing had a significantly higher probability of needing refinement compared to those with mild spacing. A previous study compared the achieved and predicted tooth movement of maxillary first molars and central incisors with Invisalign for 30 patients. The predicted and achieved tooth movement was measured using actual pre-treatment model and virtual post-treatment model. The authors reported in their study that the control over first molar anchorage and central incisor retraction was not completely achieved. They concluded that to improve the attainment of predicted changes, the use of auxiliary anchorage devices, power ridges, attachment designs, and overcorrection should be taken into consideration [13]. These results emphasize the importance of careful patient selection and treatment planning and encourage the orthodontists to overcorrect in space closure using clear aligners particularly in cases with more significant spacing issues and longer treatment time. The overcorrection of space closure might decrease the need for refinements and optimize treatment outcomes.

It is important to acknowledge some limitations of this study. The main limitation of the study is that treatment with clear aligners relies on patient compliance. The statistical power and generalizability of the findings may have been influenced by several factors. Firstly, the study had a relatively small sample size. Additionally, the majority of participants were female (83.6%; N=46). Secondly, the sampling technique employed was convenience sampling. Lastly, all participants were recruited from a single center. Therefore, caution should be exercised when interpreting the results. Our study possesses several strengths. Firstly, the treatment evaluated in this study was administered by a single orthodontist, ensuring consistency in the application of the treatment protocol. Additionally, several demographic and clinical/orthodontic variables were conducted, allowing for a more comprehensive analysis. However, given the need for further research in this area, future prospective studies with rigorous methodology and adequate sample sizes are necessary to provide additional insights into the effectiveness of clear aligners.

## Conclusions

In conclusion, this study contributes to the growing body of evidence supporting the effectiveness of Invisalign clear aligners in orthodontic treatment. The findings highlight the impact of certain demographic and orthodontic variables, such as the severity of initial spacing and the length of treatment, on treatment outcomes and the need for refinement. Clinicians should consider these factors when selecting patients for Invisalign treatment and developing treatment plans. Further research with larger sample sizes and diverse populations is needed to validate these findings and explore additional factors that may influence treatment outcomes with Invisalign.
